# Extensive Cystic Disease of the Liver in a Pig

**Published:** 1883-10

**Authors:** Wm. Osler

**Affiliations:** McGill College, Montreal


### CASE DEPARTMENT.

### I.—EXTENSIVE CYSTIC DISEASE OF THE LIVER IN A PIG.

BY WM. OSLER, M.D.

My attention was directed by the inspector of one of the city abattoirs to the liver of a hog which had been killed a day or two previous. The entire organ was occupied by simple cysts varying in size from a marble to an orange, uniformly distributed through the substance and filled with a clear fluid. At the edges and between the cysts portions of hepatic tissue, of a deep olive green color, could be seen. The organ was enormously enlarged and weighed forty pounds, about ten times the normal weight. Unfortunately the health officer had given orders for the specimen to be kept until the next meeting of the health committee, and when available was too decomposed for further dissection.

General cystic disease of the liver is a rare affection. In man very few cases of it are on record, and one or two of these have been associated with cystic kidneys. (Wilks Path. Soc. Trans. vol. vii.) The mode of origin is unknown; possibly in both liver and kidney the condition is always congenital. The hog in this case was fairly nourished, but presented great abdominal distension.

McGill College, Montreal.

